# Function and mechanism by which interferon regulatory factor-1 inhibits oncogenesis

**DOI:** 10.3892/ol.2012.1051

**Published:** 2012-11-28

**Authors:** FEI-FEI CHEN, GUAN JIANG, KERUI XU, JUN-NIAN ZHENG

**Affiliations:** 1 Laboratory of Biological Cancer Therapy, Xuzhou Medical College, Jiangsu 221002, P.R. China;; 2 Department of Biology, Wake Forest University, Salem, NC 27106, USA

**Keywords:** IRF-1, oncogenesis, cell cycle, apoptosis, immune response

## Abstract

The present review focuses on recent advances in the understanding of the molecular mechnisms by which interferon regulatory factor (IRF)-1 inhibits oncogenesis. IRF-1 is associated with regulation of interferon α and β transcription. In addition, numerous clinical studies have indicated that IRF-1 gene deletion or rearrangement correlates with development of specific forms of human cancer. IRF-1 has been revealed to exhibit marked functional diversity in the regulation of oncogenesis. IRF-1 activates a set of target genes associated with regulation of the cell cycle, apoptosis and the immune response. The role of IRF-1 in the regulation of various types of human tumor has important implications for understanding the susceptibility and progression of cancer. In addition, an improved understanding of the role of IRF-1 in the pathological processes that lead to human malignant diseases may aid development of novel therapeutic strategies.

## Contents

1. Introduction
2. IRF-1 in human cancer
3. Tumor suppressive activity of IRF-1
4. Regulation of the cell cycle
5. Regulation of apoptosis
6. Regulation of immune response
7. Conclusion

## 1. Introduction

**Figure 1.**
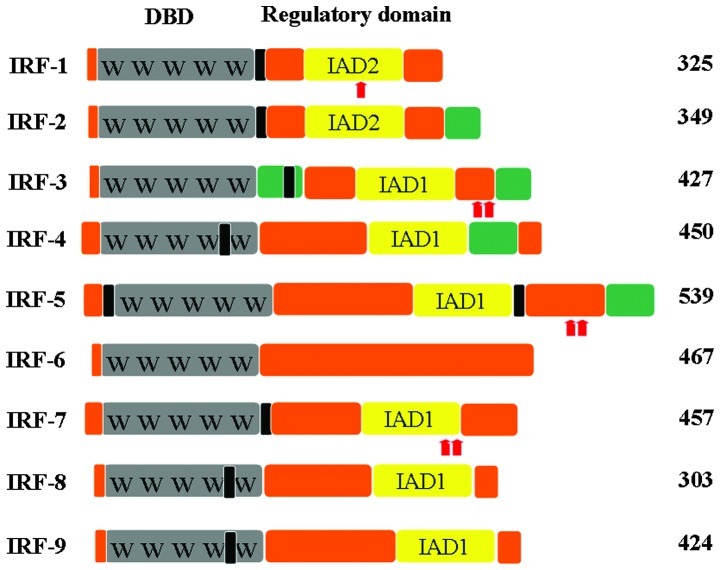
Structure of IRFs. All IRFs contain an amino-terminal DNA binding domain (DBD) that is characterized by a series of five well-conserved tryptophan-rich repeats (grey) and a regulatory domain (yellow). The DBD forms a helix-turn-helix domain and recognizes a DNA sequence similar to the IFN-stimulated response element. The majority of IRFs also contain an IRF-association domain (IAD) of type 1 (IAD1) or type 2 (IAD2). Specific IRFs contain repression domain(s) (red) and a nuclear-import signal(s) (black). For IRF-1, 3, 5 and 7, the mark of red arrows show that activity depends on phosphorylation. The number of amino acids of each IRF is indicated. IFN, interferon; IRF, IFN regulatory factor.

The original identification of the first member of the interferon (IFN) regulatory factor (IRF) family, IRF-1, was followed by additional studies on type I IFN and IFN-inducible genes in the IFN system, which is referred to as ‘the IRF kingdom’. The mammalian IRF family of transcription factors is comprised of 9 members: IRF-1, -2, -3, -4/PIP/LSIRF/ICSAT, IRF-5, -6, -7, -8/ICSBP and -9 (1,2). IRFs contain an amino-terminal DNA binding domain (DBD) which is characterized by a series of five well-conserved tryptophan-rich repeats (3,4). The DBD forms a helix-turn-helix domain and recognizes a DNA sequence similar to the IFN-stimulated response element (A/GNGAAANNGAAACT) (5). The carboxyl-terminal regions of IRFs are less well-conserved and mediate interactions with additional IRF members, transcription factors and cofactors, conferring specific activities upon each IRF (Fig. 1) (5,6). Although IRFs were first characterized as transcriptional regulators of type I IFNs and IFN-inducible genes, more recent studies indicate that this family is important for the regulation of oncogenesis beyond the IFN system (6).

IRF-1 was the first member of the IRF family to be isolated by virtue of its affinity to specific DNA sequences in the IFN-β promoter (7). The molecule is markedly induced by IFN, double-stranded RNA (dsRNA), cytokines and specific hormones. Induced IRF-1 activates target genes leading to inhibition of cell proliferation and stimulation of cell apoptosis (5). IRF-2 binds to the same DNA sequences as IRF-1 but downregulates or blocks the activation of IRF-1 target genes. Accumulating evidence indicates that IRF-1 and IRF-2 have antioncogenic and oncogenic potentials, respectively (6). A correlation between IRF-4 and oncogenesis has also been reported in Epstein-Barr virus (EBV)-transformed lymphocytes and HTLV-1-induced leukemogenesis (8,9). Notably, IRF-4 alone is not sufficient for oncognesis in transgenic mice overexpressing IRF-4 in lymphocytes. IRF-4 may regulate cellular growth by targeting pro-apoptotic IRF-5 during EBV transformation (10,11). In addition, IRF-8 has been revealed to exhibit antitumor activity through direct control of cell growth, differentiation and apoptosis and modulation of antitumor immunity (12). IRF-6 may also act as a tumor suppressor via its interaction with maspin, a tumor suppressor gene (13). An additional member of the IRF family, IRF-7, mediates the EBV transformation process in the pathogenesis of EBV-associated lymphomas indicative of oncogenic properties (14). Together, these observations indicate that the IRF family is important for regulation of oncogenesis.

Accumulating evidence indicates that deletion or inactivation of the IRF-1 gene may be a critical step in the development of specific types of human cancer. A previous study on IRF-1 identified a tumor suppressive function demonstrating the importance of additional investigation of IRF-1 in oncogenesis regulation. In the present review, we summarize the contribution of IRF-1 to regulation of oncogenesis. In addition, current hypotheses with regard to the molecular mechanisms by which IRF-1 regulates oncogenesis are discussed. IRF-1 may suppress tumor proliferation by inhibiting the cell cycle, reducing susceptibility to transformation by oncogenes or inducing apoptosis, development of natural killer (NK) cells and differentiation of Th1 and CD8^+^ T cells. Review of the current literature on IRF-1 is likely to improve understanding of the role of this molecule in the pathological processes that lead to human malignant diseases and may provide novel therapeutic strategies.

## 2. IRF-1 in human cancer

Previous clinical studies have indicated that loss of IRF-1 may affect development of specific forms of human cancer. This hypothesis was developed following the observation that the IRF-1 gene maps to the chromosomal region 5q31.1 (15). Deletion of this region is one of the most frequently observed cytogenetic abnormalities in leukemia or preleukemic myelodysplastic syndrome (MDS) (16). Loss of one IRF-1 allele has also been reported in esophageal and gastric cancers (17–19). Loss of heterozygosity analysis at the IRF-1 locus of sporadic breast cancer demonstrated frequent loss of heterozygosity at the IRF-1 gene, which may induce low IRF-1 mRNA expression. These observations imply a correlation between the IRF-1 gene and poor clinical outcome in breast cancer (20). Notably, a polymorphism in the IRF-1 gene was detected at a higher frequency in human breast cancer cell lines than in the general population and is more frequent in African-American than Caucasian individuals of European-ancestry (21). A significant difference in genotype distribution between these populations was identified (21). Collectively, these studies indicate that genetic alterations of IRF-1 are important for the development of specific types of human cancer.

In addition to genetic alterations of IRF-1 genotypes, several additional mechanisms by which IRF-1 may be inactivated in cancer have been reported. For instance, nucleophosmin, a putative ribosome assembly factor commonly overexpressed in myeloid leukaemia cells, binds to and inhibits the function of IRF-1 (22,23). Specific splicing aberrations of the IRF-1 gene leads to loss of functional IRF-1 in MDS and leukemias (24,25). A mechanism associated with the human papilloma virus (HPV) 16-encoded E7 oncoprotein has also been reported (26). In addition, previous studies indicate that SUMOylated IRF-1 inhibits apoptosis by repression of transcriptional activity (27). Finally, numerous reports reveal low expression levels of IRF-1 mRNA in specific forms of cancer, including breast cancer and hepatocellular carinoma (28,29). To develop an improved understanding of the role of IRF-1 in human cancer, additional studies must be performed to clarify the mechanism by which IRF-1 regulates oncogenesis.

## 3. Tumor suppressive activity of IRF-1

The tumor suppressor-like activity of IRF-1 was previously demonstrated by oncogenic transformation assays in which the activated c-Ha-Ras, a single oncogene, was introduced to and transformed IRF^−/−^ MEF cells (30). Wild-type MEF cells are known to require at least two oncogenes to undergo transformation. In addition, conditions under which activated Ras paradoxically inhibits cell growth of myeloid cells have been demonstrated to be involved with IRF-1 and the induction of p21^WAF1/CIP1^(31). A previous study revealed that ectopic expression of IRF-1 suppresses the malignant properties of cancer cell lines and oncogene-transformed cell lines *in vitro* and *in vivo*(29).

In contrast to other tumor suppressor genes, loss of IRF-1 alleles alone rarely induces tumor development, however, IRF-1 deficiency was previously demonstrated to markedly enhance tumor predispositions caused by the expression of a c-Ha-Ras transgene or nullizygosity of the transformation-related protein 53 (Trp53) gene (32). This accelerated tumor development may not be due to the discussed immunological disorders caused by the absence of IRF-1 (33). Therefore, IRF-1 belongs to a class of tumor susceptibility genes whose loss in combination with other genetic alterations significantly increases the incidence of developing tumors.

The mechanisms by which IRF-1 mediates tumor suppression are not well understood. Several IRF-1 induced genes that exert growth-inhibitory and promote apoptosis effects, including 2′,5′-oligo(A) synthetase (34), indoleamine 2,3-dioxigenase (35), RNA-dependent protein kinase (PKR) (36), p21^WAF/CIP1^(37), Lox (38), angiotensin type II receptor (39), TNF-related apoptosis-inducing ligand (TRAIL) (40), caspase-1 (41), -7 (42) and -8 (43) may be associated with inhibition of proliferation, induction of apoptosis, stimulation of the immune response and reversion of the transformed phenotype (Table I). Among them, Lox is important for biogenesis of connective tissue matrices and is identical to the Ras recision gene, which is associated with in the reversion of Ras-transformed NIH3T3 cells by preventing the activation of nuclear factor κ-light-chain-enhancer of activated B (44). Aberrant expression of Lox leads to tumorigenesis and tumor progression. However, this molecule is also a potential downstream mediator for the tumor-suppressive activity of IRF-1. By contrast, an additional study demonstrated that growth of the Ras^+^ myc-induced transforming phenotype in soft agar is not altered by overexpression of Lox, but is suppressed by IRF-1 expression (45).

More recent studies have identified additional IRF-1 target genes. The tumor suppressor activity of IRF-1 has been associated with downregulation of cyclin D1 (46) and survivin (47). A ChIP-chip approach performed by Frontini *et al*(48) revealed a novel role for IRF-1 in the regulation of the DNA interstrand crosslink damage response. IRF-1 regulates ATP-dependent RNA helicase (BRIP1), a component of the Fanconi anemia/BRCA DNA repair pathway and a newly identified breast cancer susceptibility gene (49,50). The association between IRF-1 and BRIP1 further validates the importance of IRF-1 as a tumor susceptibility gene (48). In addition, hydroxyprostaglandin, pleiomorphic adenoma gene-like 1, Ras association domain family 5, a kinase (PRKA) anchor protein 12 and deleted in colorectal cancer have been identified as IRF-1 target genes with tumor suppressor activities (48). Further studies to identify the role of these individual genes in the antitumor activity of IRF-1 must be performed.

## 4. Regulation of the cell cycle

The involvement of IRF-1 in cell cycle regulation has been studied extensively and its tumor suppressive activity may be explained, at least in part, by its cell cycle checkpoint function. In fact, the expression of IRF-1 appears to be regulated throughout the cell cycle. IRF-1 mRNA expression is markedly elevated in NIH3T3 cells subjected to serum-induced cell cycle progression; however, it rapidly decreases as the serum-induced cell cycle continues, suggesting that IRF-1 is involved in cell cycle regulation (51). Several studies have provided insight into the mechanism by which IRF-1 inhibits the cell cycle.

The growth inhibitory effects of IRF-1 may be mediated by stimulation of antiproliferative gene transcription. As with p53, transcriptional induction of the gene encoding p21^WAF1/CIP1^ by γ irradiation has also been identified to be regulated by IRF-1 (37). In DNA-damaged cells, IRF-1 protein levels were elevated via an Ataxia telangiectasia mutated-dependent increase in mRNA expression and protein half-life, acting on the p21^WAF1/CIP1^ promoter region containing the IRF-1 and p53-binding sites to induce a G_1_ cell cycle-specific arrest (37).

Moreover, additional genes or secreted factors induced by IRF-1 may also cause cell growth inhibition, including 2′,5′-OAS, whose products activate the mRNA-degrading enzyme RNase L (34). Xie *et al* established a casual series of events that functionally connect the antiproliferative effects of IFNs with the IRF-1-dependent suppression of the CDK2 gene, which encodes a key regulator of the G_1_/S phase transition. Although IRF-1, -2, -3 and -7 have all been demonstrated to activate IRF-1-responsive reporter genes, only IRF-1 inhibits CDK2 gene transcription (52).

The IRF-1-induced enzymes, including lysyl oxidase and indoleamine 2,3-dioxygenase, may lower the biosynthetic capacity of the cell by enhanced degradation of rate-limiting precursors (35,38). PKR is important for the regulation of cell proliferation and exerts antigrowth activities by IFN-inducible genes, including IRF-1 (53).

Specific signal pathways are also vital for the regulation of growth activity. For instance, the Janus kinase and signal transducer and activator of transcription (JAK-STAT) pathway may be an IRF-1 target for growth regulation at the transcriptional level (54). However, STAT1 is known to function upstream of IRF-1 and regulate IRF-1 promoter expression. This mechanism is currently hypothesized to involve IRF-1 upregulation in response to IFN induction through STAT1. Newly synthesized IRF-1 may in turn activate expression of STAT1, resulting in positive feedback regulation of IRF-1 expression (55).

## 5. Regulation of apoptosis

Apoptosis is an additional mechanism used to control cell number in tissues and eliminate individual cells that threaten the host’s survival. IRF-1 is associated with apoptosis induced by DNA damage or other stimuli (56). Wild-type MEFs, when introduced with an activated oncogene, i.e., c-Ha-Ras, undergo apoptosis instead of cell cycle arrest when treated with anti-cancer drugs or ionizing radiation. Apoptosis is a hallmark of tumor suppression and is dependent, in this case, on IRF-1 and p53 (30).

However, DNA damage-induced apoptosis in mitogenically activated mature T lymphocytes is dependent on IRF-1 but independent of p53 (57,58). Bowie *et al* demonstrated that IRF-1 is critical for the promotion of p53-independent apoptosis in acutely damaged basal-type human mammary epithelial cells, providing evidence that loss of IRF-1 is a short-term marker of early basal-type breast cancer risk (59). Pizzoferrato *et al* identified that ectopic expression of IRF-1 protein results in downregulation of survivin protein expression that is independent of p53 and promotes breast cancer cell death (47). In addition, IRF-1 binds to distinct sites in the promoter and upregulates expression of PUMA, a p53-upregulated modulator of apoptosis that activates apoptosis by the intrinsic pathway. PUMA has also been identified to function in a p53-independent manner (60). Therefore, IRF-1 induces apoptosis by the intrinsic pathway, independent of the extrinsic pathway, by upregulation of PUMA. However, in thymocytes, apoptosis is dependent on p53 but not on IRF-1. Thus, IRF-1 and p53 regulate DNA damage-induced apoptosis cooperatively and independently, depending on the type and differentiation stage of the cell. Notably, gatekeeper of apoptosis activating proteins-1, a transcriptional activator of IRF-1 and p53, has a proapoptotic activity (61).

Caspases are unique proteases that comprise an activation cascade downstream in the apoptosis mechanism. IRF-1 has been demonstrated to directly mediate IFN-γ-induced apoptosis via activation of caspase-1 gene expression in IFN-γ-sensitive ovarian cancer cells and other cancer cells (62). Furthermore, IRF-1 is known to activate caspase-8 expression in response to IFN-γ/STAT1 signaling, a component of the events that sensitize cells for apoptosis (63). Caspase activity assays are used to determine the overexpression of wild-type IRF-1 or dominant negative IRF-1 in breast cancer cells. Thus, IRF-1 controls apoptosis through caspase-8 in breast cancer cells. These observations are consistent with the hypothesis that IRF-1 regulates apoptosis through caspase-8 in breast cancer cells (64). Moreover, RNA interference experiments also indicated that IRF-1 and -2 are associated with constitutive caspase-8 expression in neuroblastoma cells (65). In addition, Tomita *et al* demonstrated that IRF-1 is important for IFN-γ mediated-enhancement of Fas/CD95-mediated apoptosis through the regulation of DEVD-CHO-sensitive caspases, most likely caspase-7 (66).

Moreover, specific genes or signaling molecules may be involved in IRF-1-regulated apoptosis. TRAIL signaling is critically involved in immune surveillance against tumor development. IFN-γ enhances the anticancer activities of TRAIL through IRF-1 (67). Promoter mapping, chromatin immunoprecipitation and RNA interference reveal that retinoid-induced IRF-1 is required for TRAIL induction by retinoic acid (RA) and IFN-γ (40).

## 6. Regulation of immune response

IRF-1 regulates the expression of a number of genes whose products are central to innate and adaptive immunity, indicating that IRF-1 may provide a link between the two systems. IRF-1 induces transcription of various genes involved in the first reaction to viral invasion, including PKR and 2′,5′-OAS (36,34). IRF-1 also binds MyD88 and is modified by currently unidentified signaling molecule(s) to migrate into the nucleus and induce genes encoding IFN-β, iNOS and IL-12p35 (68).

In addition to the functions assigned to IRF-1 in differentiated immune cells, previous studies have revealed roles for IRF-1 in the development of various immune cells. IRF-1 affects the development and function of NK cells. Analysis of the spleen and liver of Irf-1^−/−^ mice demonstrates a reduction in NK cell counts and function (69). Reduced IRF-1 selectively affects bone marrow stromal cells that constitute the microenvironment for NK cell development. IRF-1 does not affect NK progenitors (70). However, IRF-1 in stromal cells is required for transcription of the gene encoding IL-15, which is essential for NK cell development (71).

IRF-1 also regulates dendritic cell subset development. Mice lacking IRF-1 have reduced numbers of mature CD8^+^ T cells, despite normal maturation of CD4^+^ T cells in the thymus and peripheral lymphoid organs (72,73). Low molecular weight protein-2, antigen processing-1 and major histocompatibility complex I are decreased in Irf-1^−/−^ thymic stromal cells (73–75), however, the defect in CD8^+^ T cell development does not reside in the thymic environment but is instead due to a thymocyte-intrinsic defect during differentiation from immature to mature CD8^+^ T cells (76). TCR stimulation induces IRF-1 expression in immature thymocytes, while Irf-1^−/−^ thymocytes are defective in TCR-mediated signal transduction. Therefore, IRF-1 may regulate genes in developing T cells that are crucial for signal transduction in the thymus and in lineage-specific differentiation of CD8^+^ T cells (76).

Moreover, IRF-1 promotes the differentiation of Th1. T-cells from Irf-1^−/−^ mice fail to mount Th1 responses and instead exclusively undergo Th2 differentiation *in vitro*(77). IRF-1 has also been identified to be involved in the differentiation of Th1 combined with HPV E7 (78). However, compromised Th1 differentiation is associated with defects in multiple cell types, including impaired production of the p40 subunit of interleukin-12 (IL-12p40) by macrophages and hyporesponsiveness of CD4+ T cells to IL-12, which is essential for Th1 differentiation (69,77). IRF-1 regulates the expression of genes encoding iNOS induced by IFN-γ. iNOS catalyzes the production of nitric oxide, a short-lived volatile gas important in the effector phase of the Th1 response (79). In essence, IRF-1 is indispensable for the differentiation of Th1.

The discussed observations demonstrate that tumor surveillance by the immune system is impaired by loss of IRF-1. Therefore, IRF-1 may function as a ‘systemic gatekeeper’ involved in the protection of the host against exogenous mutagens that may lead to caricinogenesis.

## 7. Conclusion

The previous studies discussed reveal that the IRF-1 transcription factor is involved in the regulation of tumor suppression and oncogenesis. However, the mechanisms underlying IRF-1-mediated tumor suppression and oncogenesis remain undefined.

The high frequency of mutated or rearranged IRF-1 in several cancer types indicates its importance in oncogenesis. Additional studies demonstrate that IRF-1 exhibits tumor suppressor activities in a number of human tumors. IRF-1 domains and post-translational modifications may affect the function of the transcription factor. IRF-1 activates a set of target genes responsible for inhibition of the cell cycle and induction of apoptosis and may regulate DNA repair. Therefore, in order to gain an improved understanding of the role of IRF-1 in oncogenesis, new targets of IRF-1 must be identified which are critical in the cascade of events involved in oncogenesis.

IRF-1 is a unique member of the IRF transcription factor family as it functions in the regulation of the innate and adaptive immune systems, linking the immune response and oncogenesis together. Elucidation of the intricate gene network operation associated with regulation of host defense and the function of IRF-1 in this network is not currently understood fully. However, the multiple functions of IRF-1 imply that IRF-1 may be suitable as a selective gene with an engineered delivery system for biotherapies of various types of cancer and autoimmune diseases.

## Figures and Tables

**Table I.** IRF-1 target genes.

Gene	Role	Reference
IFN-α/β	Antiviral response	5,7
p21^WAF/CIP1^	Cell cycle	38
p53	Apoptosis	21
PKR	Cell cycle	37
2′,5′-OAS	Antiviral response	53
Lox	Inhibition of cell transformation	39
Angiotensin type II receptor	Apoptosis	40
TRAIL	Apoptosis	41
Caspase-1	Apoptosis	42
Caspase-7	Apoptosis	43
Caspase-8	Apoptosis	44, 69
PUMA	Apoptosis	64
BRIP1	N/A	49
BARD1	N/A	49
HPGD	Metabolic process	49
PLAGL1	Anti-proliferation	49
RASSF5	Cell adhesion	49
AKAP12	Signal transduction	49
IL-15	NK cell development	76
iNOS	Th1 differentiation	73
LMP-2	CD8^+^ T cell development	80
TAP-1	CD8^+^ T cell development	79
MHC-I	CD8^+^ T cell development	78
IL-12p40	Th1 differentiation	74

IFN, interferon; IRF, IFN regulatory factor; PKR, RNA-dependent protein kinase; 2′,5′-OAS, 2′,5′-oligo(A) synthesis; Lox, lysol oxidize; TRAIL, TNF-related apoptosis-inducing ligand; PUMA, p53 unregulated modulator; BRIP1, ATP-dependent RNA helicase; BARD1, BRCA1-associated ring domain protein 1; HPGD, hydroxyprostaglandin; PLAGL1, pleiomorphic adenoma gene-like 1; RASSF5, Ras association domain family 5; AKAP12, a kinase (PRKA) anchor protein 12; LMP-2, low molecular weight protein-2; TAP-1, transporter associated with antigen processing-1; MHC-I, major histocompatibility complex I; IL, interleukin.
